# Correction to: Comparison of minimally invasive Ivor Lewis esophagectomy and left transthoracic esophagectomy in esophageal squamous cell carcinoma patients: a propensity score-matched analysis

**DOI:** 10.1186/s12885-020-06999-8

**Published:** 2020-06-25

**Authors:** Qi Wang, Zixiang Wu, Tianwei Zhan, Shuai Fang, Sai Zhang, Gang Shen, Ming Wu

**Affiliations:** Department of Thoracic Surgery, 2nd Affiliated Hospital, School of Medicine, Zhejiang University, 88 JieFang Rd, Hangzhou, 310009 China

**Correction to: BMC Cancer 19, 500 (2019)**

**https://doi.org/10.1186/s12885-019-5656-7**

Following publication of the original article [1], the authors identified an error on the acknowledgements section of the article. The acknowledgments declaration should state the following:

**Acknowledgments**

We express our thanks to the following people for giving valuable information and suggestion for this article: Dr. Chai Huiping (The First Affiliated Hospital of Anhui Medical University, Anhui, China), Dr. Yu Guangmao (Shaoxing People’s Hospital, Shaoxing, China).
